# Bacterial–fungal interactions: connections and consequences

**DOI:** 10.1042/EBC20250037

**Published:** 2026-09-02

**Authors:** Rebecca A. Hall, Katherine J. Baxter

**Affiliations:** 1 School of Natural Sciences, Biosciences, University of Kent, Canterbury CT2 7NJ, U.K.; 2 Plant Science Group, School of Molecular Biosciences, College of Medical Veterinary and Life Sciences, University of Glasgow, Glasgow, G12 8QQ, UK.

**Keywords:** Bacterial-fungal interactions, interkingdom interactions, Microbial communities, Microbial-host interactions, microbiology

## Abstract

In nature, microorganisms exist in multispecies microbial communities containing bacteria, fungi, archaea, and viruses. The organisation, behaviour, and ecological impact of these communities are very much defined by the various interactions between bacteria and fungi within the community, with physical associations, chemical communication, metabolic exchange, and genetic regulation collectively shaping how these interkingdom communities assemble, adapt, and influence their hosts and habitats. Methods of interaction are widely shared across the microbiota of plants, animals, and the built environment; however, interkingdom microbial communities have environmentally specific outcomes, meaning it is critically important to understand bacterial–fungal interactions (BFIs) within the host or environmental context. With recent advances in BFI analysis now providing increasingly detailed resolution of BFIs and their function in the dialogue between interkingdom microbial communities and their growth environment, we can now gain better insight into these fundamental processes. In the present mini-review, we detail the main BFIs observed in these interkingdom microbial communities, and their implications in the context of plant, human, and the health of the built environment. We also discuss tools and methodologies for their analysis and potential use in the development of microbially derived technologies to improve health and well-being. Finally, we endorse the perspective that interkingdom microbial communities should be considered as structured, interdependent networks with analogy to multicellular organisation.

## Introduction

Microbial communities are shaped by a dense web of interactions that extend across kingdoms, with bacterial–fungal interactions (BFIs) representing one of the most influential drivers of community structure and function [1]. Rather than acting in isolation, bacteria and fungi engage in coordinated physical, chemical, and metabolic exchanges that collectively determine how multispecies consortia behave within their ecological or host environments [2,3]. Although interkingdom microbial communities do not constitute tissues in the classical biological sense, they exhibit a degree of structural integration and functional interdependence that invites comparison with multicellular systems [4]. This holistic perspective is now applied in synthetic ecology, where simple communities are used to expand understanding in microbial community dynamics, BFIs, and microbe–host interactions [5].

Within the community, BFIs are broadly categorised into four mechanistic domains: physical associations, chemical communication, metabolic cross-feeding/nutrient competition, and genetic/regulatory outcomes. These mechanistic foundations manifest differently across ecological contexts. In plants, synergistic interactions between arbuscular mycorrhizal fungi (AMF) and plant growth promoting rhizobacteria (PGPR) enhance nutrient acquisition and plant immunity, while antagonistic interactions suppress fungal phytopathogens [6]. In the human body, BFIs contribute to both homeostasis and disease, influencing outcomes in the gut, lung, oral cavity, and vaginal microbiome [7,8]. In the built environment, interkingdom communities shape biofilm resilience, air quality, and occupant health [9].

## Bacterial–fungal interactions are ubiquitous

All life on Earth is underpinned by microbially driven processes with BFIs ubiquitous across a wide variety of ecological niches [1]. These interactions have specific roles, functions, and behaviours within their host or environment that modulate or influence their surrounding habitat [3,10,11].

### Plant-associated interkingdom interactions

In terms of plant-associated BFIs, there are two main groups of associated microbiotas in which these can be found: those residing within the soil and rhizosphere (soil region adjacent to the roots), and those within the phyllosphere (aerial part of the plant). These microbiomes are exposed to different host environments, with phyllosphere microorganisms being exposed to wider ranges of abiotic and environmental factors, while those inhabiting the rhizosphere are exposed to a variety of plant exudates retained by the soil [12,13]. In the rhizosphere, AMF interact with PGPR to promote plant health and facilitate uptake of nutrients from the soil in return for plant photosynthates [14,15]. Examples of such interactions include those between the PGPR *Bacillus subtilis*, and AMF species *Rhizophagus clarus* and *Claroideoglomus etunicatum* that promote increased levels of photosynthetic pigments, reduce stress levels, and increase plant biomass through enhanced phosphorous uptake [16,17]. PGPR*/*AMF consortia interactions can also enrich soil nitrogen [18] and increase rhizosphere microbial diversity [19], offering further plant health benefits through the recruitment of other nutrient-enriching and protective microorganisms [20]. These PGPR/AMF interactions are critical for plant immunity, offering biocontrol of both bacterial and fungal plant pathogens. For example, synergism between the Gram-negative PGPR *Paraburkolderia graminis* and AMF *Funneliformis mosseae* increases plant immune responses and prevents attack by the bacterial pathogen *Xanthomonas translucens* in wheat [21], and other multi-PGPR and AMF consortia protect date palms against soil-borne fungal disease caused by *Fusarium oxysporum* [22].

However, synergism within rhizosphere BFI is a double-edged sword, with other interactions promoting microbial growth, survivability, and virulence that compromise plant health and augment infection. Fungi-associated bacteria can increase resistance of fungi to community-synthesised antimicrobial molecules, reducing the impact of naturally occurring biocontrol mechanisms within the microbiome [23,24]. In terms of virulence, some fungal phytopathogens rely on bacterial partners for toxin production [25]. Furthermore, bacterial association with fungal hyphae allows bacterial niche expansion, increasing opportunity for greater bacterial–bacterial interactions within the soil community and facilitating contact-dependent killing of competitors [26]. This could lead to overgrowth of bacterial phytopathogens or increase the reservoir of human and animal opportunistic pathogens within the soil [27].

In contrast with the rhizosphere, BFIs within the phyllosphere are less well understood. Phyllosphere microorganisms face abiotic challenges such as UV exposure, wind, and rain [12,28] and have higher turnover as plant tissue returns to the ground as leaf litter [28], making their communities more transient. Studies of BFIs within the phyllosphere are dominated by antagonistic interactions by bacteria against fungal phytopathogens (plant pathogens), such as suppression of the fungal wheat pathogen *Zymoseptoria tritici* by *Bacillus velezensis* through the production of the antifungal lipopeptides fengycin and baccilomycin [29] and the ability of multiple bacterial components of the coffee plant phyllosphere to inhibit the growth of the leading coffee rust fungal phytopathogen, *Hemileia vastatrix*, the mechanism of which is yet to be identified [30].

### Human-associated interkingdom interactions

A vast effort has gone into profiling the human microbiome and mycobiome, providing a wealth of information on the microbial composition of each niche and how these are impacted by disease and therapeutic intervention [31–35]. However, these studies do not directly look at microbial interactions, but more advanced studies combining next generation sequencing with metabolomics, tissue biopsies, and *in vitro* interactions are shedding light on the role of polymicrobial interactions in human health.

The gastrointestinal tract contains the largest diversity of microbes and is therefore a key niche for polymicrobial interactions. In inflammatory bowel disease, the inflammatory environment of the gut results in reduced bacterial diversity and enhanced colonisation by *Candida albicans* [36]. Increased abundance of *C. albicans* can also impact the bacterial composition of the microbiome through utilisation of oxygen, to promote the colonisation of anaerobic bacteria like *Clostridium difficile* [37]. On the other hand, BFI interactions in the gut can be directly antagonistic, with *Serratia marcescens* killing *C. albicans* via type 6 secretion system effectors [38], and *Saccharomyces cerevisiae* preventing the attachment and colonisation of enterotoxigenic *Escherichia coli* [39].

**Figure 1:**
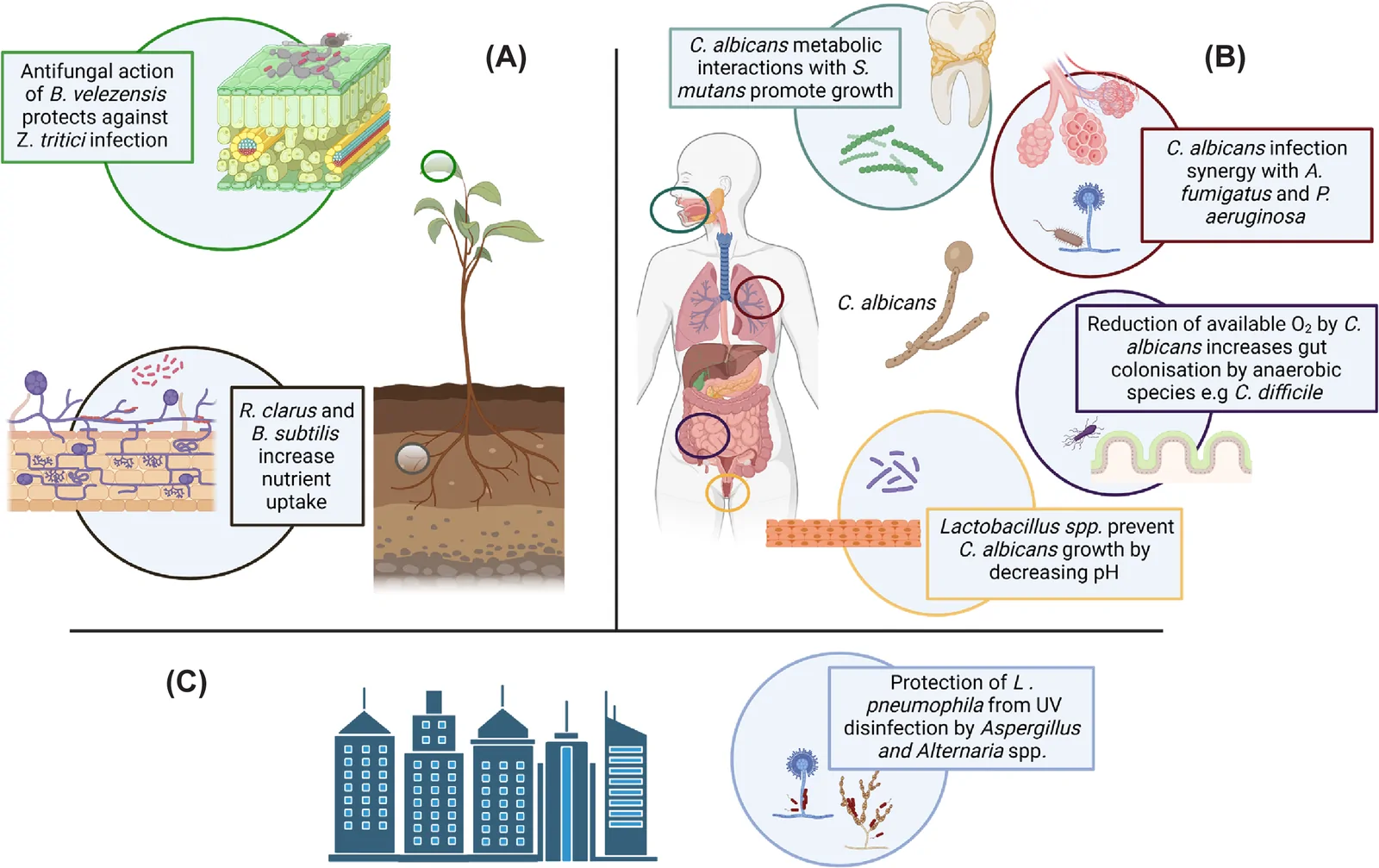
Comparative niche map Top left panel (**A**) Plant niches. Example rhizosphere interactions include synergistic interactions to promote plant growth and antagonistic interactions to prevent fungal infection [16,29] Top right panel (**B**) Human niches. Examples of *C. albicans* interactions across four different body niches showing synergy in oral plaque formation [131,132], the CF lung [42] and gut microbiome dysbiosis [37], and antagonism preventing infection in the vaginal microbiome [47]. Bottom panel (**C**) The built environment. Co-occurrence of fungal species increases the resistance of *Legionella* to UV treatment, a commonly used disinfection method [67]. Created in BioRender. https://BioRender.com/0zzx06t

The cystic fibrosis (CF) lung provides a unique environment for polymicrobial interactions. By adolescence, CF patients are chronically colonised with *Pseudomonas aeruginosa* [40,41] with the co-occurrence of fungi like *C. albicans* and *Aspergillus fumigatus* associated with a greater decline in lung function and poor disease prognosis [42]. In the oral cavity, dental plaque is a polymicrobial community providing ample opportunity for direct and indirect interkingdom interactions. Similar interactions occur on medical devices like voice prothesis where attachment and morphogenesis of *C. albicans* promotes bacterial adhesion and polymicrobial biofilm formation, reducing the functionality and lifetime of the device [43,44]. In the vagina, Lactobacilli are important to maintain the pH and health of the vaginal mucosa, with several Lactobacillus species inhibiting the morphogenesis of *C. albicans* and displaying antifungal activity against *C. albicans* [45–47]. Figure 1 summarises these different BFI interactions across plant, human, and built environment niches.

In addition to affecting disease progression, polymicrobial interactions have implications for antimicrobial therapy. In biofilms, fungal carbohydrates contribute to the extracellular matrix and sequester antimicrobial agents, preventing them from reaching their target [48–50]. On the other hand, bacterial secreted molecules can heighten fungal intracellular ROS production, which synergises with the mode of action of some antifungals enhancing drug efficacy [51]. For some interactions, there are contrasting reports on the impact on antimicrobial resistance, suggesting that the local environment probably has a role to play in determining outcomes [52].

### Bacterial–fungal interactions in the built environment

This interplay between bacteria and fungi extends beyond plants, humans, and other organisms to our built environment, the microbiome of which is dynamically exchanged with microorganisms from the population who frequent the building, structure, or facility [53]. Co-occurrence of bacterial and fungal species can be observed in healthcare facilities [54], in homes and other non-clinical settings [55,56], wastewater facilities [57], and even on the International Space Station (ISS) [58–60]. Although BFIs are not well characterised in the built environment beyond the acute care setting [61,62], their role in sick building syndrome, where building occupants experience acute health conditions such as headaches, cold or allergy symptoms, skin rashes, and fatigue while in the building, is recognised [56,63]. It is not unreasonable to expect similar outcomes in terms of robust biofilm formation and increased resistance to antimicrobials, cleaning, and disinfectant treatments [64–67] occurring in other structures and facilities outside of the healthcare setting. Indeed, study of homes and offices shows increased resistance to UV disinfection by *Legionella pneumophilia* in the presence of *Aspergillus* and *Alternaria* [67] (Figure 1).

Within buildings and structures, bacterial–fungal dialogue within the microbial community also influences the production of microbial volatile organic compounds (VOCs). Modulated by both interkingdom interactions and the type of building material on which the community is established [68,69], VOCs are thought to contribute to the increased prevalence of allergic conditions [70]. Furthermore, in water-damaged buildings, combinations of microbial toxins and bacterial and fungal cell wall components are known to trigger proinflammatory responses in components of the immune system [71]. Interkingdom interactions can further impact health through our water processing and distribution systems. Bioaerosols containing bacterial and fungal pathogens, several of which are known to form interkingdom communities, are liberated during processing at wastewater treatment plants [57], and bacterial and fungal species present in our water supply form multispecies biofilms within pipe networks [72]. In addition, these biofilms may also act as a reservoir for viruses [73,74]. Biofilms in water supplies are also a serious concern in closed-loop systems where water reclamation is essential, such as on the ISS [75]. Interestingly, spaceflight is a closed loop, high-stress environment with a profoundly different biophysical environment to that which BFIs have evolved under, posing a unique setting for interrogating their functionality and role in community behaviour in the future [76].

## Mechanisms of interaction

BFIs are orchestrated through a diverse suite of physical, chemical, metabolic, and genetic mechanisms that shape how microbes coexist, compete, and interact within their community.

### Physical associations within interkingdom communities

Bacteria and fungi interact physically. This direct interaction within interkingdom communities facilitates the distribution of bacteria within the growth environment [77], increases the proximity of both partners to metabolites synthesised by the other [78,79], and any chemical alterations to the microenvironment driven by microbial behaviour [80,81]. These physical interactions also offer mechanical feedback of shear stress and flow alterations within the surrounding environment to the multispecies community [82].

Direct physical interaction is mediated by Lifshitz–Van der Waals and acid–base interactions [83], and adhesins, surface proteins found on both bacteria and fungi that drive interactions with the host, abiotic surfaces, and other microorganisms, and facilitate biofilm formation [84–86]. For example, in the pleiomorphic fungus *C. albicans*, there are several adhesin and adhesin-like proteins [87], with the agglutin-like-sequence (Als) family of proteins predominating microbial interactions [88–90]. Their importance is highlighted by their conservation in non-*albicans* species [91,92]. In Gram-positive bacteria, microbial surface components recognising adhesive matrix molecules (MSCRAMMs) and other surface proteins like the antigen I/II family in *Streptococcus mutans* facilitate interactions [93,94], whereas in Gram-negative bacteria fimbrial, and nonfimbrial adhesins are key [95]. Adhesins and their involvement in BFIs are well characterised in *C. albicans*. Known examples include *C. albicans* and *Streptococcus gordonii* interaction through Als3 and SspB [88] and *C. albicans* and *Staphylococcus aureus* through *S. aureus* binding to *C. albicans* Als3 [89], although there is evidence of the role of other Als family members [96] and possibly MSCRAMM involvement [97]. Gram-negative bacteria and fungal interactions can be orchestrated through binding of bacterial adhesins with the fungal cell wall component, mannan. Examples of this binding capacity include type I fimbriae of *E. coli*, which mediate interactions with *S. cerevisiae* [98], and the mannose-specific lectin Msl of the vaginal commensal *Lactobacillus planatarum*, which binds *C. albicans* and *S. cerevisiae* [99]. Other BFIs of Gram-negative species occur through type IV pili, as seen with *P. aeruginosa* and *C. albicans* [100], and the type VI secretion system [38]. In the rhizosphere, bacterial–fungal physical interactions go beyond surface interactions with the presence of a variety of different bacterial species residing within both hyphae and chlamydospores of soil-dwelling fungi [101–103].

Fungal hyphae also play a pivotal role in the movement of bacteria. For example, physical attachment of bacteria to fungal hyphae can allow dissemination through host tissues, as observed with *C. albicans* and *S. aureus* co-infections [77,104], and an equivalent mechanism exists in the rhizosphere, with bacterial biofilms on AMF hyphae co-migrating with hyphae [105]. Indeed, the use of hyphae as a means of bacterial dissemination is a common occurrence in the rhizosphere, with the term ‘fungal highway’ being used to describe the movement of microorganisms via hyphal means [106].

### Bacterial–fungal chemical communication

Fungi and bacteria co-ordinate and communicate through the release of secreted molecules; these can either be soluble molecules or secondary metabolites, or VOCs released into the atmosphere.

Quorum sensing molecules (QSMs) are released in a cell-density-dependent manner and, once at threshold concentrations, regulate and co-ordinate the expression of virulence factors and biosynthesis of secondary metabolites [107,108]. The most extensively studied QS interaction occurs between *P. aeruginosa* and *C. albicans*. Here, bacterial secretion of 3-oxo-C12 homoserine lactone inhibits morphogenesis [109] and biofilm formation [110], while inducing apoptosis in *C. albicans*. However, this relationship is not one sided. *Candida albicans* secretes the QSM farnesol to promote biofilm dispersal, but farnesol also inhibits PQS production [111], siderophore production [112], and biofilm formation of *P. aeruginosa*, with farnesol emulsions being developed as antibiofilm therapies for several ESKAPE pathogens [113–115]. The diffusible signal factor (DSF) family of QSMs are widely used by Gram-negative bacteria to regulate adaptation and virulence. In terms of interkingdom signalling, DSF molecules produced by *Stenotrophomonas maltophilia, Streptococcus mutans*, and members of the *Burkholderia cerpacia* complex have been shown to inhibit biofilm formation, morphogenesis, and adhesion of *C. albicans* [113,116–119]. Mechanistically, *Burkholderia* DSF inhibits morphogenesis via inhibition of the fungal cAMP-PKA pathway in a similar vein as the fungal QSM farnesol. Given the structural similarity between the DSF family and farnesol, and that dodecanol inhibits cAMP signalling in *C. albicans* [120], it is likely that inhibition of cAMP signalling is central to the mechanism of all members of the DSF molecules.

Microbial VOCs encompass a broad range of compounds including aldehydes, alcohols, and ketones [121]. In agricultural settings, bacteria-produced VOCs play an important role in controlling plant fungal pathogens by inhibiting mycelial growth through reduced membrane integrity, increased ROS, and lipid peroxidation. Sulphur-containing VOCs produced by *Collimonas* have broad antifungal activity, with N-acetylglucosamine enhancing VOC production [122], suggesting that bacterial sensing of chitin plays an important role. VOCs produced by *Streptomyces setonii* inhibit the growth and germination of *Ceratocystis fimbriata*, the causative agent of black spot disease in sweet potatoes, via inhibition of ribosome biogenesis and disruption to cell wall synthesis [123]. These VOCs also activate plant defences, increasing disease resistance [123], suggesting a two-pronged approach to combating *C. fimbriata* infections.

### Occurrences of metabolic cross-feeding and nutrient exchange

In all contexts, microbes compete with each other for space and nutrients. Mammals limit the availability of key micronutrients (i.e., iron, zinc) through nutritional immunity, inhibiting microbial growth [124], with growing evidence of similar systems in plants [125]. However, fungi and bacteria have evolved mechanisms to compete with the host for these essential micronutrients. For example, *P. aeruginosa* secretes high affinity siderophores (i.e., pyoverdine) to compete for iron [126]. These siderophores restrict the accessibility of iron to other opportunistic pathogens like *Rhizopus microsporus* inhibiting their growth [127]. In addition to competition, *P. aeruginosa* can also work synergistically with the microbiome by degrading mucins, providing alternative metabolites like succinate and acetate to support microbial growth [128]

In the gut, *C. albicans* and *Enterococcus faecalis* undergo a cross-feeding cycle, whereby *C. albicans* enhances citrate production that is then broken down by *E. faecalis* to formate and subsequently detoxified by *C. albicans* [129]. However, the role of this cross-feeding in commensalism and/or pathogenicity is not known. Other important examples of metabolic cross-feeding occur in dental plaque. Here, *C. albicans* cannot metabolize dietary sucrose, limiting its growth in the oral cavity, but *S. mutans* breaks down sucrose into glucose and fructose, providing a rich nutrient source to promote *C. albicans* proliferation [130]. *Candida albicans* then promotes *S. gordonii* glycoside hydrolase expression, enabling the bacterium to breakdown glycoproteins under nutrient-limited environments like saliva [131]. The continued proliferation of *C. albicans* increases oxygen consumption that subsequently promotes the growth of anaerobic bacteria [132] and therefore the development of dental plaque (Figure 1).

### Genetic and regulatory dynamics within communities of bacteria and fungi

**Figure 2:**
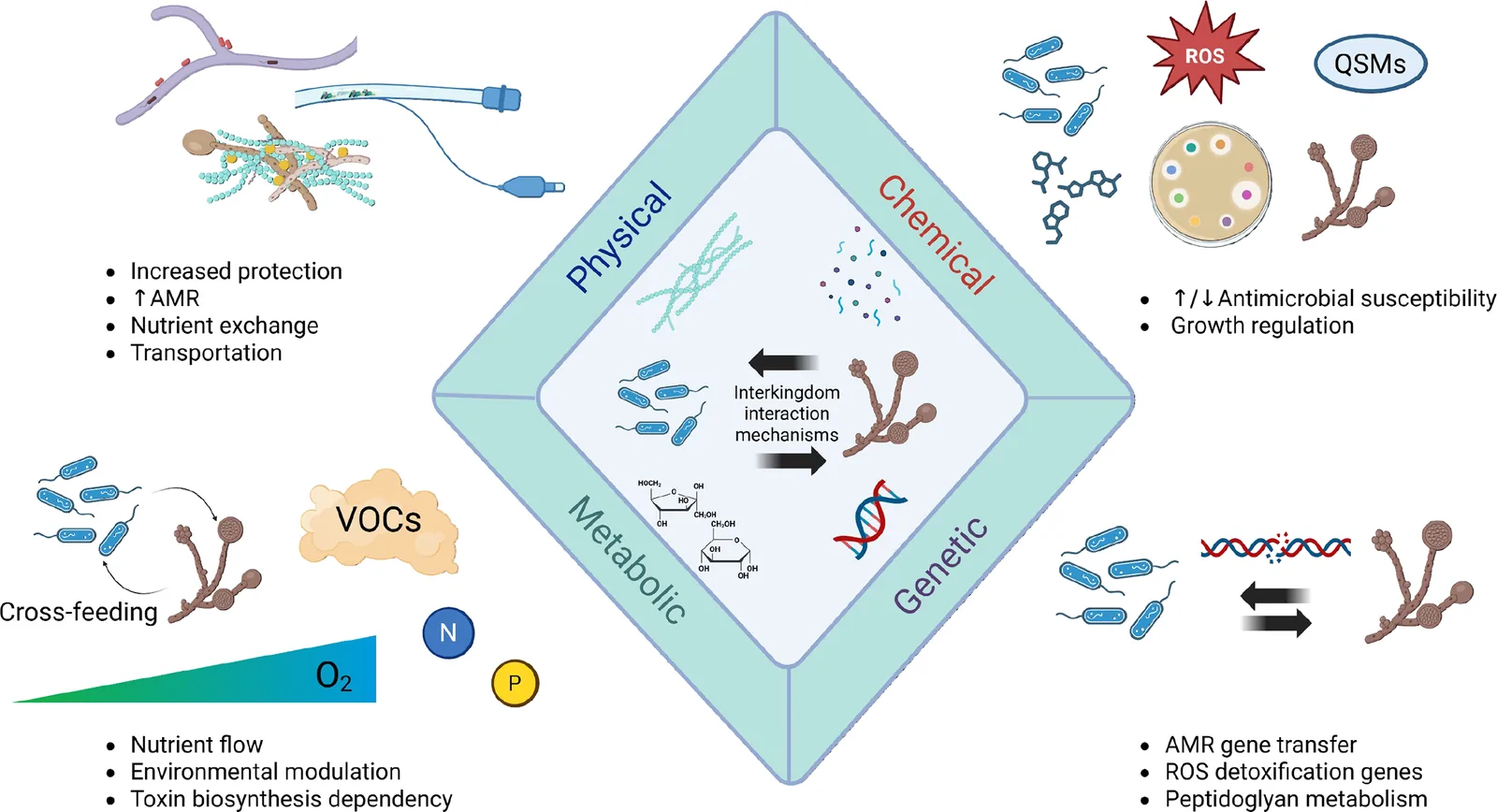
Mechanisms of BFI interactions A central hub illustrates the forms of interaction driving BFIs. Physical, chemical, metabolic, and genetic processes operate in combination across ecological niches. Examples of resulting outcomes are detailed below each interaction type. Created in BioRender. Baxter, K. (2026) https://BioRender.com/1hl7kz6

Horizontal gene transfer (HGT) enables genetic exchange between species, diversifying the genetic pool and frequently promotes the acquisition of genes involved in virulence and antimicrobial resistance. Genetic exchange in bacteria and fungi is common, and there are reports of interkingdom genetic exchange. For example, antibiotic resistance genes have been identified in fungal genomes [133] along with enzymes involved in ROS detoxification and peptidoglycan metabolism [134]. Endosymbiotic bacteria play an important role in interkingdom HGT [135], although experimental validation is required to ensure predictions based on sequencing are not a result of contamination. Aquatic environments appear to be hot spots for interkingdom HGT, likely due to the nature of the environment and possibility for polymicrobial biofilm formation. Figure 2 illustrates the mechanisms of bacteria–fungal interactions.

## Tools and approaches to study bacterial–fungal interactions

**Figure 3:**
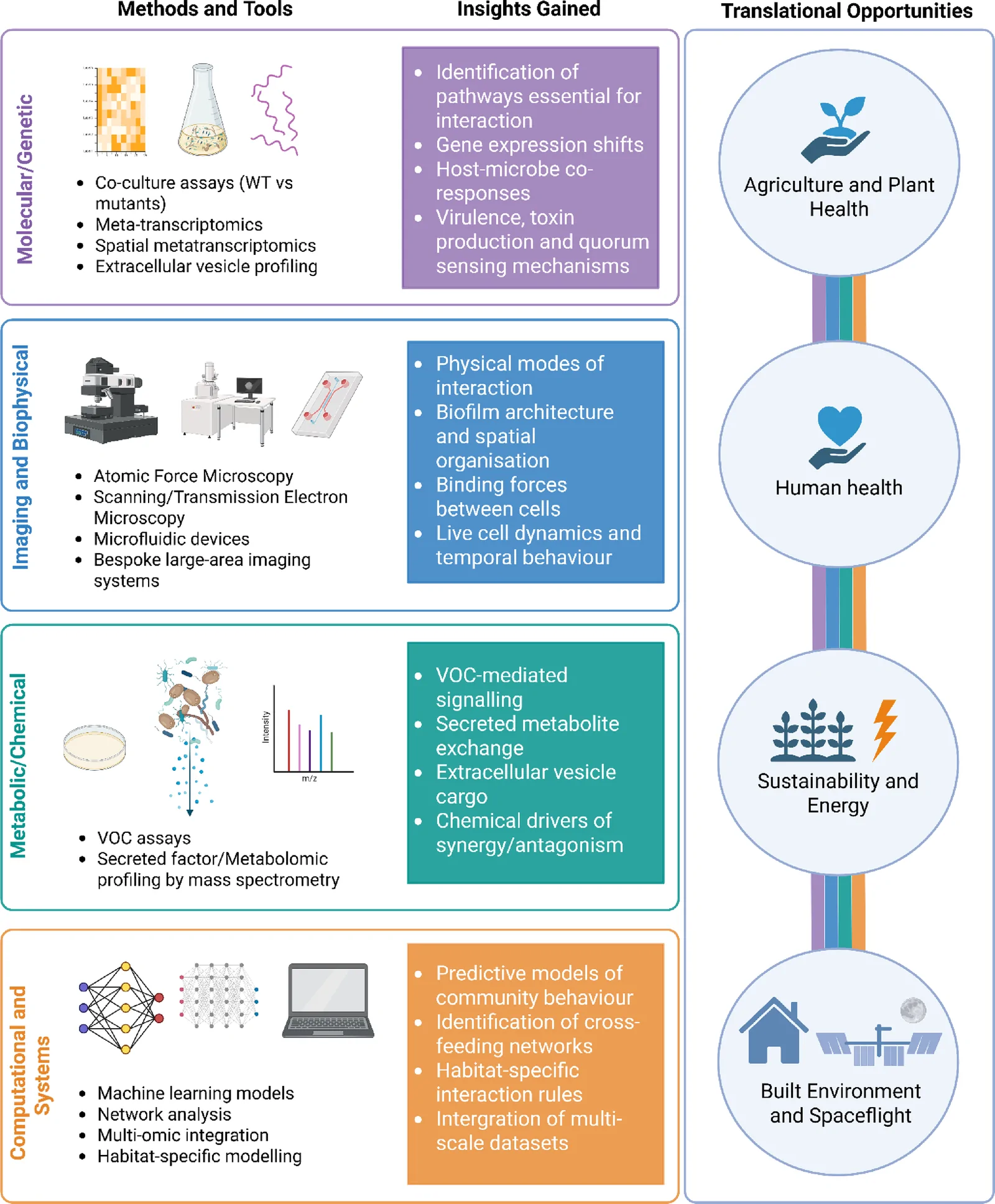
From tools to translation Multiscale experimental and computational approaches reveal the molecular, physical, metabolic, and systems-level processes underpinning BFIs. Their combined use can aid development of new technologies across four broad areas: agriculture and plant health: microbial consortia for increased yield; biocontrol against pathogens; bioremediation of agrichemicals; and soil microbiome engineering. Human health: microbiome-based therapeutics; extracellular vesicle-derived diagnostics; drug delivery; drug engineering; and antimicrobial material engineering. Sustainability and energy: microbial fuel cells; waste processing and circular economy; and biodegradation pathways. Built environment and spaceflight: material-driven microbiome design, advanced material design, and biofouling control. Created in BioRender. Baxter, K. (2026) https://BioRender.com/ov2wlmq

A variety of different tools and approaches are employed for the study of BFIs (Figure 3). Co-culturing of commonly associated bacteria and fungi and comparison of their wild-type and mutant strains can be used to identify pathways and metabolic processes critical to their interactions [136–138], and microfluidic devices are employed to test responses to metabolites and signalling molecules [139] or utilised for microscopical analysis of interactions [140]. Furthermore, methods that employ physical separation of bacteria and fungi but maintain exposure to VOCs, such as the overlapping plate assay, can identify VOCs involved in bacterial/fungal interactions when they are coupled to mass spectrometry methods [141]. Similarly, the release of extracellular vesicles during BFI can also be identified though metabolomics [142,143]. This methodology has also been mooted as an alternative to invasive diagnostics for the identification of pathogens and their metabolic state in infection [144].

In terms of microscopical methods, there are number of different multiscale imaging methodologies that can provide detailed information on BFIs. At the nanoscale, atomic force microscopy can analyse binding forces between cells [145], and both scanning electron microscopy and transmission electron microscopy can provide visualisation of bacterial–fungal cellular interactions [146]. Confocal scanning laser microscopy does not have the same resolution as EM methods but permits imaging of both live cells and fixed cells, with live cells visualised using either endogenously encoded fluorescent proteins or live-compatible stains [147], whereas fixed cells are imaged with fluorescent antibody labelling [148]. At the mesoscale, large macrostructures produced by BFIs can be visualised over a large field of view, either through stitch-and-tile methodologies [149] or by bespoke imaging systems [97,150].

Approaches that generate big datasets and apply systems biology approaches are powerful tools for interrogating BFIs. Meta-transcriptomics can reveal changes in gene expression in response to interacting partners [151] and spatial meta-transcriptomics can identify host responses in tandem with those of their associated microbial consortia [152]. Computationally, machine learning algorithms and network analyses can model habitat-specific details and infer complex interactions from multi-omic data, to give further insight into bacterial–fungal dynamics [153,154].

The limitations in all these studies lie in the fact that each provides a snapshot of BFIs under those given conditions. Infectious and ecological microenvironments influence and define microbial community organisation and behaviour [15,52,155], and so what we observe may not truly reflect interkingdom interactions in their natural environment. However, since all microorganisms are governed by biophysical limitations surrounding nutrient acquisition and survival, comparative analyses of multiple datasets can be useful for the identification of these underlying processes within microbial communities [156,157].

## Translational opportunities and future directions

Understanding of the complex dynamics of BFIs and viewing the co-ordinated, systems level behaviour of interkingdom microbial communities through the lens of multicellularity offers significant opportunity for the development of microbially driven technologies for plant, human, and building health (Figure 3). In agriculture, antagonistic and synergistic interactions are already being employed to increase plant yield and mitigate infection. Commercial formulations of AMF and PGPR consortia are available for both household and farm use [158] and both bacterial and fungal-derived biocontrol methods against key plant pathogens are in development world-wide [159]. BFIs may also provide a route for bioremediation of agrichemicals from farmland [160,161] and generate energy in microbial fuel cells [162] supporting sustainability. With regard to human health, augmentation of various microbiomes to prevent or ameliorate disease is a focus for research and commercialisation, with therapeutic targeting of the gut and skin microbiome [163,164], and the manipulation of BFIs for drug delivery [165]. In the built environment, building design and material use can influence microbial colonisation and BFIs [166]. As with plant and human health, biocontrol methods leveraging antagonistic BFIs are also applicable in the built environment [167].

Other aspects of translational opportunity from BFIs include the development of methods that target the molecular systems controlling microbial physiology and community behaviour. Disrupting quorum sensing and secondary metabolite production can impact biofilm formation, virulence factor expression, and microbial community structure [109–111,168], and siderophore and other nutrient uptake systems identified through analyses of cross-feeding events may also be viable targets [127,169–171]. However, to truly explore these technologies we must employ standardised methodologies and assay *in situ* systems that faithfully recreate the microenvironments and wider ecology in which interkingdom communities thrive.

## Conclusion

BFIs are a multifaceted interplay of molecular processes that collectively shape the behaviour of interkingdom microbial communities. Cumulatively, this mirrors aspects of multicellular organisation and offers a useful conceptual lens for understanding how interkingdom consortia assemble and function. Comparison across ecological niches demonstrates that although specific outcomes of BFIs are shaped by local microenvironments, the underlying principles governing attachment, communication, nutrient exchange, and regulatory adaptation are widely conserved. This suggests that universal processes can be identified through comparative analysis of diverse interkingdom microbial communities. This systems-level perspective provides a strong foundation for translational innovation, enabled by emergent technologies and meta-analyses that allow researchers to capture the complexity of BFIs within their native ecological and infectious microenvironments. By integrating mechanistic depth with comparative ecological insight, we can better understand the fundamental processes that govern interkingdom community dynamics and translate this knowledge into strategies that support plant, human, and environmental health.

## Summary

- BFIs are fundamental to microbial community structure and ecosystem outcomes across humans, plants, and built environments.
- Core mechanisms of physical contact, signalling, metabolism, and gene regulation are conserved, although outcomes depend on local microenvironments.
- BFIs drive both beneficial and detrimental effects, from the promotion of plant growth to human disease and biofilm resilience.
- Advances in analysis methods provide insight into interkingdom interactions, with future work aiming to harness BFIs for biocontrol, microbiome engineering, and therapeutic innovation.

## Open Access

Open access for this article was enabled by the participation of the University of Glasgow in an all-inclusive Read & Publish agreement with Portland Press and the Biochemical Society under an agreement with JISC.

## Abbreviations

AMF: arbuscular mycorrhizal fungi
BFI: bacterial–fungal interaction
DSF: diffusible signal factor
HGT: horizontal gene transfer
MSCRAMMs: microbial surface components recognising adhesive matrix molecules
PGPR: plant growth promoting rhizobacteria
VOC: volatile organic compounds
ROS: reactive oxygen species
QS: quorum signalling
PQS: *Pseudomonas* quinolone signal
ESKAPE: *Enterococcus faecium, Staphylococcus aureus, Klebsiella pneumoniae, Acinetobacter baumannii, Pseudomonas aeruginosa*, and *Enterobacter* species
EM: Electron Microscopy
